# Glucose Prediction under Variable-Length Time-Stamped Daily Events: A Seasonal Stochastic Local Modeling Framework

**DOI:** 10.3390/s21093188

**Published:** 2021-05-04

**Authors:** Eslam Montaser, José-Luis Díez, Jorge Bondia

**Affiliations:** 1 Instituto Universitario de Automática e Informática Industrial, Universitat Politècnica de València, Camino de Vera, s/n, 46022 València, Spain; emontase@gmail.com (E.M.); jldiez@isa.upv.es (J.-L.D.); 2 Centro de Investigación Biomédica en Red de Diabetes y Enfermedades Metabólicas Asociadas (CIBERDEM), Instituto de Salud Carlos III, 28029 Madrid, Spain

**Keywords:** type 1 diabetes, glucose prediction, seasonal local models, Fuzzy C-Means

## Abstract

Accurate glucose prediction along a long-enough time horizon is a key component for technology to improve type 1 diabetes treatment. Subjects with diabetes might benefit from supervision and control systems that accurately predict risks and trigger corrective actions early enough with improved mitigation. However, large intra-patient variability poses big challenges to glucose prediction. In previous works by the authors, clustering and local modeling techniques with seasonal stochastic models proved to be efficient, allowing for good glucose prediction accuracy for long prediction horizons. Continuous glucose monitoring (CGM) data were partitioned into fixed-length postprandial time subseries and clustered with Fuzzy C-Means to collect similar behaviors, enforcing seasonality at each cluster after subseries concatenation. Then, seasonal stochastic models were identified for each cluster and local predictions were integrated into a global prediction. However, free-living conditions do not support the fixed-length partition of CGM data since daily events duration is variable. In this work, a new algorithm is provided to overcome this constraint, allowing better coping with patient’s variability under variable-length time-stamped daily events in supervision and control applications. Besides predicted glucose, two real-time indices are additionally provided—a crispness index, indicating good representation of current glucose behavior by a single model, and a normality index, allowing for the detection of an abnormal glucose behavior (unusual according to registered historical data). The framework is tested in a proof-of-concept in silico study with ten patients over four month training data and two independent two month validation datasets, with and without abnormal behaviors, from the distribution version of the UVA/Padova simulator extended with diverse sources of intra-patient variability.

## 1. Introduction

Diabetes represents one of the major health challenges of the 21st century. According to [1], 463 million people have diabetes worldwide in 2019, and this figure is estimated to rise to 700 million by 2045. Type 1 diabetes (T1D), the most severe kind, affects about 10% of people with diabetes. In T1D, β-cells in the pancreas are destroyed by an autoimmune response. These cells secrete insulin, which is an essential hormone needed to maintain blood glucose (BG) concentrations in a narrow range (70–140 mg/dL). Insulin promotes the transport of glucose into the cell and its lack translates into elevated BG (hyperglycemia). Thus, T1D treatment consists in insulin replacement by means of subcutaneous administration of insulin analogs in order to avoid the long-term complications due to hyperglycemia. This is carried out either by multiple daily injections with insulin pens or continuous infusion through insulin pumps. However, overdosing of insulin can provoke too low BG concentration (hypoglycemia), with severe consequences if untreated, including comma and death. Good BG control is challenging, and requires a lot of effort from patients, who must frequently monitor their BG levels and take dosing decisions to avoid hyperglycemia and hypoglycemia. Nowadays, glucose concentration can be measured in a quasi-continuous way by using continuous glucose monitoring (CGM) devices, which consist of an electrochemical sensor measuring interstitial glucose at the subcutaneous space and estimating BG, which is transmitted periodically (every 5 min) to an external receiver [2].

Accurate glucose prediction along a given time horizon is a key component in current diabetes technology. Patient monitoring systems must raise warnings of risks enough time ahead to successfully prevent extreme hyperglycemic and, especially, hypoglycemic episodes [3]. In sensor-pump integrated systems, predicted glucose levels can trigger actions by the insulin pump, like automatic insulin infusion suspension to mitigate impending hypoglycemia [4]. Glucose prediction is also an important feature in artificial pancreas (AP) systems, that is, closed-loop glucose control systems where a control algorithm governs the insulin pump from CGM data [5,6]. AP research has been intense in the last decade. A first commercial system was launched in late 2017, a second one in early 2020, and many more are on the way [7,8]. Glucose prediction can be an integral part of the control algorithm itself, such as in Model Predictive Control-based AP systems [9]. Therefore, high-reliability glucose prediction models have the potential to significantly improve diabetes management as part of a monitoring system, integrated systems or an AP.

A huge number of alternatives for BG prediction in T1D have appeared in the literature, such as, among others, linear empirical dynamic models, multivariate nonlinear regression techniques, extended Kalman filters, data mining or artificial intelligence approaches [10]. In [11,12], seasonal stochastic local models are introduced for the first time in the field aiming at improved glucose prediction under high intra-patient variability. The rationale lies on the fact that observed behaviors in past similar scenarios can help improving prediction at the present moment. Seasonal stochastic time series models such as SARIMA (Seasonal AutoRegressive Integrated Moving Average), or SARIMAX (SARIMA including eXogenous variables), are meant to capture regular periodical patterns in time series data [13]. Although seasonality is not a priori a characteristic of glucose data in T1D, it can be enforced partitioning historical CGM data into fixed-length time subseries and concatenating them, after a clustering process collecting similar behaviors based on a given similarity measure. Then, seasonal stochastic models can be identified for each cluster and their predictions integrated to provide a global glucose prediction. Seasonal models have exhibited relatively higher postprandial BG prediction accuracy for long prediction horizons [11], including challenging scenarios with variety of meals and exercise sessions [12]. In this latter case, SARIMAX models were used including insulin infusion and energy expenditure from a wearable as exogenous inputs.

In the above works, a concatenation of fixed-length postprandial time subseries driven by mealtime was considered, from controlled studies. Although this can be appropriate to test performance and concepts underlaying the new prediction methodology, its application in free-living conditions do not support fixed-length partition of CGM data, as required to enforce seasonality. Daily events such as mealtime and night periods happen at non regular times and have variable lengths. In this work, algorithms are modified to overcome this constraint, allowing to cope with patient’s variability under variable-length time-stamped events, and paving the way to its application in supervision and control in T1D. CGM data is partitioned into a collection of “event-to-event” time subseries whose length is regularized (unified) by adding fictitious blank samples where needed to enforce seasonality. This imposes the use of new clustering algorithms able to deal with these blank samples, which correspond to missing (incomplete) data. Main events are mealtimes and night periods, although other events could be considered as long as a timestamp exists, such as reported hypoglycemia treatments or exercise sessions. Besides predicted glucose, two real-time indices are additionally provided indicating *crispness* of the prediction (degree of representation of current glucose behavior by a single model), and *normality* of the observed behavior (degree of representation by registered historical data), allowing for the detection of patient’s abnormal states (unusual or not seen before in historical data), which might trigger conservativeness in the insulin therapy for the sake of safety.

## 2. Materials and Methods

### 2.1. Data Overview

Three datasets were generated for the ten adults virtual cohort in the educational version of the UVA/Padova simulator [14], which was extended with an exercise model [15], and diverse sources of intra-patient variability:

- Training dataset: Four-month data was generated for the ten patient cohort following open loop therapy (basal insulin and ratios provided in the simulator were used). The 15-15 rule was used to treat hypoglycemia (15 g of carbohydrates were administered when glucose went below 70 mg/dL, and repeated if after 15 min hypoglycemia was still present). A nominal day with three meals of 40, 90 and 60 g of carbohydrates at 7:00, 14:00 and 21:00 h was considered. Actual mealtime and carbohydrate intake varied around nominal values following a normal distribution with a standard deviation of 20 min for mealtime, and with a 10% coefficient of variance for meal size. Although no study on dietary habits was performed to set this amount of variability on meal size, a 10% coefficient of variance managed to challenge this in silico study with a wide enough variety of meal sizes, ranging from 20 to 120 g, distributed around the chosen nominal values. Meal absorption dynamics was changed at each meal by randomly selecting one of meal model parameter sets available from the simulator, resulting in faster or slower meal absorptions especially due to nonlinearities in gastric emptying emulating a slow down of carbohydrates absorption produced by fats [14]. Additionally, meal absorption rate ($k_{abs}$) varied with a uniform distribution in ±30%, and carbohydrate bioavailability (*f*) in ±10% around selected nominal values. Carb counting errors by the patient were considered with a uniform distribution between −30% and +10%, following results in [16] where a trend to meal underestimation is reported. Insulin absorption pharmacokinetics ($k_{d}$, $k_{a1}$, $k_{a2}$) varied in ±30%, according to the intra-patient variability reported in [17]. Circadian variability of insulin sensitivity ($V_{mx}$, $K_{p3}$) was considered with variations in ±30% around nominal sensitivity, reproducing changes in basal insulin requirements in the adult population reported in [18]. The reader is referred to [14] for the model and parameters details. Finally, missed boluses were sporadically generated with a minimum separation of 14 days between them in order to better mimic imperfect data collection. These postprandial periods were later excluded to compose the training dataset from collected data.
- Validation dataset 1 (“normal”): Two-month data was generated for the 10-patient cohort for the same scenario as data used for training. Thus, events and variability in this validation dataset is expected to be well represented in training data (it contains “normal” data).
- Validation dataset 2 (“abnormal”): Two-month data was generated for the 10-patient cohort adding to the above scenario missed boluses (once per week), and three moderate intensity (50% VO2max) exercise sessions per week (on Tuesday, Thursday and Saturday). Nominal duration of the exercise duration was 60 min, and nominal start time at 18:00 h. Actual duration and start time changed at each session following a normal distribution with coefficient of variance of 10% for duration and standard deviation of 20 min for start time. Since training data does not contain missed boluses and exercise, these events are expected not to be well represented in training data (the dataset contains “abnormal” data).

In all the above datasets, besides CGM data, time stamps and labels for the following events were generated: *Breakfast, Lunch*, *Dinner*, *Night* and *Hypoglycemia treatment*. The *Night* event was considered to start 6 h after dinner. All rescue carbs administered in a hypoglycemia treatment were gathered in a single *Hypoglycemia treatment* event, starting at the time of the first rescue carb intake. This will be represented by the set of time-ordered pairs

(1) $$E:=\{\left(t_{e_{i}},L_{e_{i}}\right)\;|\;i=1,\ldots ,n_{e}\},$$

where $t_{e_{i}}$ is the timestamp for the *i*-th event, with $t_{e_{i}}<t_{e_{i+1}}$, $L_{e_{i}}$ is the label of the *i*-th event, $L_{e_{i}}\in \left\{\mathrm{Breakfast},\mathrm{Lunch},\mathrm{Dinner},\mathrm{Night},\mathrm{Hypoglycemia treatment}\right\}$, and $n_{e}$ is the number of events.

In a clinical context, assumption of existence of time stamps for meals and night period is a mild one. Mealtime can be automatically extracted from insulin pump information (bolus calculator), or from the recently marketed smart pens in a multiple daily injections therapy. The start of a night period is defined algorithmically and no input is necessary from the patient. Hypoglycemia occurrence can be detected from CGM data and only confirmation of rescue carbs intake by the patient would be needed. However, this information in not critical since the amount of carbs is not needed. In the absence of this confirmation, assumption of treatment of any hypoglycemia occurrence might be assumed.

### 2.2. Enforcing Seasonality: CGM Data Partitioning

As a first step, seasonality must be enforced prior to building seasonal stochastic local models from the training data set. Provided that the starting time and duration of events in the CGM historical data is variable between event instances, the original CGM time series data is partitioned into a set of “event-to-event” time subseries from the reported timestamps and labels in the set E. A single partition can be considered if event labels are neglected, or multiple partitions can be generated according to some grouping of event labels. This latter option has the advantage of building prediction models more specific to well differentiated events (for instance a night compared to a meal), although it requires events labeling. A compromise solution is adopted here, with consideration of three partitions—meals, night and hypoglycemia treatment. No difference between breakfast, lunch and dinner is needed, requiring only information about mealtime and hypoglycemia occurrence, which can be extracted automatically. Thus, consider

(2) $$E=E_{M}\cup E_{N}\cup E_{H},$$

where

(3) $$\begin{array}{ccc} E_{M} & := & \left\{\left(t_{e_{i}},L_{e_{i}}\right)\;|\;L_{e_{i}}\in \left\{\mathrm{Breakfast},\mathrm{Lunch},\mathrm{Dinner}\right\}\right\} \end{array}$$

(4) $$\begin{array}{ccc} E_{N} & := & \left\{\left(t_{e_{i}},L_{e_{i}}\right)\;|\;L_{e_{i}}\in \left\{\mathrm{Night}\right\}\right\} \end{array}$$

(5) $$\begin{array}{ccc} E_{H} & := & \{\left(t_{e_{i}},L_{e_{i}}\right)\;|\;L_{e_{i}}\in \left\{\mathrm{Hypoglycemia}\mathrm{treatment}\right\}\}. \end{array}$$

Given the index sets

(6) $$\begin{array}{ccc} I_{M} & := & \left\{i\;|\;\left(t_{e_{i}},L_{e_{i}}\right)\in E_{M}\right\} \end{array}$$

(7) $$\begin{array}{ccc} I_{N} & := & \left\{i\;|\;\left(t_{e_{i}},L_{e_{i}}\right)\in E_{N}\right\} \end{array}$$

(8) $$\begin{array}{ccc} I_{H} & := & \left\{i\;|\;\left(t_{e_{i}},L_{e_{i}}\right)\in E_{H}\right\} \end{array}$$

and denoting by ${\left[G\right]}_{t_{0}}^{t_{1}}\left(t\right):=\left\{G\left(t\right)\;|\;t_{0}\leq t\leq t_{1}\right\}$ to the time subseries given by the segment of CGM data from $t_{0}$ to $t_{1}$, the following CGM data partitions are built:

(9) $$\begin{array}{ccc} \Pi_{M} & := & \left\{{\left[G\right]}_{t_{e_{i}}}^{t_{e_{i+1}}}\left(t\right)\;|\;i\in I_{M}\right\} \end{array}$$

(10) $$\begin{array}{ccc} \Pi_{N} & := & \left\{{\left[G\right]}_{t_{e_{i}}}^{t_{e_{i+1}}}\left(t\right)\;|\;i\in I_{N}\right\} \end{array}$$

(11) $$\begin{array}{ccc} \Pi_{H} & := & \{{\left[G\right]}_{t_{e_{i}}}^{t_{e_{i+1}}}\left(t\right)\;|\;i\in I_{H}\}. \end{array}$$

Therefore, each CGM time subseries comprises from a given event timestamp, $t_{e_{i}}$, to the next event timestamp, $t_{e_{i+1}}$.

In order to enforce seasonality, time subseries length must be regularized. To this end, a maximum expected length for an event-to-event period must be set. In this work, the period with the maximum duration for each partition $\Pi_{M}$, $\Pi_{N}$, and $\Pi_{H}$ for the training data is computed, denoted here as $L_{M}$, $L_{N}$, and $L_{H}$, respectively. Given the time subseries ${\left[G\right]}_{t_{e_{i}}}^{t_{e_{i+1}}}\left(t\right)\in R^{l_{i}}$, then $L_{M}:=\max_{i\in I_{M}}l_{i}$, $L_{N}:=\max_{i\in I_{N}}l_{i}$, and $L_{H}:=\max_{i\in I_{H}}l_{i}$. Time subseries with length $l_{i}$ smaller than the corresponding maximum length ($L_{M}$, $L_{N}$, $L_{H}$), are expanded with blank values (“not a number”—NaN) until reaching maximum length. As a result, time subseries length is regularized and seasonality can be enforced (see Section 2.4). Remark that most of time subseries will have blanks in the final positions which should be adequately treated as missing data in the clustering and identification processes. As well, regularized length will be different for each partition $\Pi_{M}$, $\Pi_{N}$, and $\Pi_{H}$.

Other events may be present in the historical data besides the considered ones, like for instance snacks and exercise. If these events are announced by the patient or can be automatically extracted from the devices, they could be included in the list of events driving data partition. Alternatively, they can be treated as exogenous inputs, as it was the case of exercise in [12] by means of signals from a wearable. Unannounced and unmeasurable events affecting CGM will be part of the data variability, which will be adequately treated in the clustering step.

### 2.3. Enforcing Similarity: Data Clustering

Once CGM data is partitioned into event-driven glucose time subseries, data in a partition are clusterized to gather similar glucose responses. Fuzzy C-Means (FCM) clustering [19] was successfully applied in previous works to the classification of similar postprandial periods [12]. However, FCM requires data to be complete for the computation of cluster prototypes (centroids) and distance measures. Thus, FCM is not directly applicable to the problem at hand due to the presence of missing data resulting from the seasonality enforcing step described in the previous section. Therefore, the Partial Distance Strategy FCM (PDSFCM) algorithm [20] is used here to overcome CGM incomplete data. Given a data set $X=\{x_{1},x_{2},\ldots ,x_{n}\}$, $x_{i}\in R^{L}$, i=1,…,n, PDSFCM partitions data into c>1 clusters through the minimization of the objective function

(12) $$J_{m}\left(U,V;X\right)=\sum_{i=1}^{c}\sum_{j=1}^{n}u_{ij}^{m}d^{2}\left(x_{j},\nu_{i}\right),$$

where $V=(\nu_{1},\nu_{2},\ldots ,\nu_{c})$ is the vector of sought cluster prototypes $\nu_{i}\in R^{L}$, where *L* is the regularized length in the partition under consideration, that is, $L_{M}$, $L_{N}$, or $L_{H}$, *d* is a partial distance function and m∈[1,∞) is the fuzziness parameter. The following partial distance is used here [20]

(13) $$\begin{array}{ccc} d(a,b) & := & \frac{L}{L-B}\sqrt{\sum_{i=1}^{L}{\left(D_{i}\right)}^{2}} \end{array}$$

(14) $$\begin{array}{ccc} D_{i} & := & \left\{\begin{array}{cc} 0 & \mathrm{if}a_{i}\mathrm{or}b_{i}\mathrm{is}\mathrm{blank}\\ a_{i}-b_{i} & \mathrm{otherwise}, \end{array}\right. \end{array}$$

for $a,b\in R^{L}$ and *B* the number of blanks (in one vector, or the other, or both). Remark that for B=0, the above distance is the standard Euclidean distance. The partition matrix $U=[u_{ij}]$, i=1,2,…,c, j=1,2,…,n, collects the membership value of each data $x_{j}$ to each cluster *i*, where $u_{ij}\in \left[0,1\right]$, $\sum_{i=1}^{c}u_{ij}=1$, ∀j and $0<\sum_{j=1}^{n}u_{ij}<n\;\;\forall i$. In our case, the data set *X* corresponds to each partition $\Pi \in \{\Pi_{M}$, $\Pi_{N}$, $\Pi_{H}\}$ of glucose time subseries ${\left[G\right]}_{t_{e_{i}}}^{t_{e_{i+1}}}\left(t\right)$ after length regularization as described in Section 2.2.

There exists a large variety of indices to determine the optimal number of clusters. In this work, the Fukuyama-Sugeno (FS) index was used [21].

### 2.4. Local SARIMA Models

**Figure 1:**
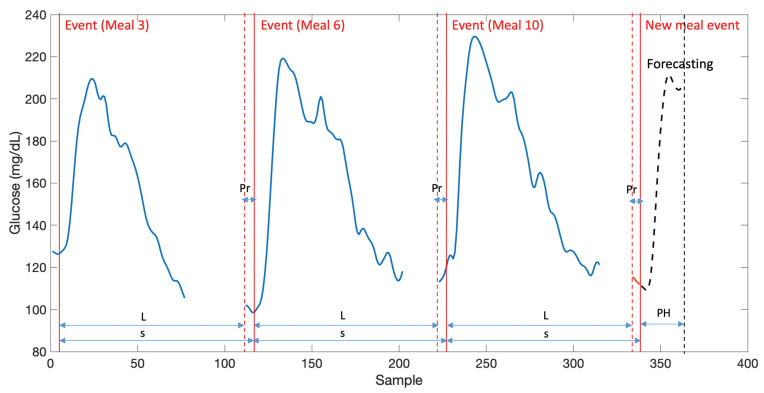
An illustrative example of a concatenated time series for seasonal stochastic local models identification. Each element in a cluster, corresponding in this case to glucose responses to meal events regularized to length *L*, are concatenated ordered according to the event time stamp, and preceded by pre-sampling data of length $P_{r}$. As a result, a seasonal time series with seasonality $s=L+P_{r}$ is obtained. Remark that meal events not necessarily are consecutive due to the clustering process.

After data in a partition are clusterized, local seasonal models are trained for each cluster. A SARIMA model, as opposed to its non-seasonal counterpart ARIMA model, includes new seasonal autoregressive (SAR) and seasonal moving-average (SMA) terms introducing dependence of an observation at time *t* on observations and stochastic errors at lags multiple of the model seasonality period *s* [22]. Combination with AR and MA components will introduce dependence on observations and stochastic errors previous to *t*, t−s, t−2s, … up to a given number of past data depending on the corresponding model component orders. Remark that this implies that CGM data before each event time is needed to properly feed the SARIMA model at $t=t_{e_{i}}$ and during the first computed samples depending on the AR order. This will be referred to as *pre-sampling data*, which length must accommodate to the maximum expected AR order, denoted as $P_{r}$. Then, a CGM time series can be built from the time-ordered concatenation of regularized-length time subseries ${\left[G\right]}_{t_{e_{i}}}^{t_{e_{i+1}}}\left(t\right)\in R^{L}$ in a cluster, preceded by the corresponding pre-sampling data, of length $P_{r}$ (see Figure 1). The resulting concatenated time series G(t) will have seasonality $s:=L+P_{r}$. Remark there will be as many concatenated CGM time series like the one in Figure 1 as number of clusters (*c*), gathering together similar behaviors. Then, a seasonal stochastic local model can be identified for each cluster.

In the absence of exogenous inputs, a local SARIMA model for a given cluster can be expressed in the form:

(15) $$\begin{array}{ccc} G(t) & = & \alpha +w(t)\\ \phi_{p}\left(z^{-1}\right)\Phi_{P}\left(z^{-s}\right)\nabla_{s}^{D}\nabla^{d}w\left(t\right) & = & \theta_{q}\left(z^{-1}\right)\Theta_{Q}\left(z^{-s}\right)\varepsilon \left(t\right),\; \end{array}$$

where G(t) is the glucose concentration at time *t*, α is a constant term (intercept), w(t) is the disturbance series, ∇ is the backward difference operator, defined by ∇w(t):=w(t)−w(t−1), *d* is the non-seasonal integration order of the time series, *D* is the seasonal integration order of the time series (in practice, *d* and *D* are usually small, equal to 0, 1 or 2), $\nabla_{s}$ is the seasonal backward difference operator, defined by $\nabla_{s}w\left(t\right):=w\left(t\right)-w\left(t-s\right)$. The input ε(t) is the stochastic error following a white noise process $\varepsilon \left(t\right)∼WN\left(0,\sigma^{2}\right)$ and $\phi_{p}\left(z^{-1}\right)$, $\Phi_{P}\left(z^{-s}\right)$, $\theta_{q}\left(z^{-1}\right)$, and $\Theta_{Q}\left(z^{-s}\right)$ are polynomials in the lag (back-shift) operator $z^{-1}$ of degree *p*, *q*, *P*, and *Q*, respectively, defined as

(16) $$\begin{array}{ccc} \phi_{p}\left(z^{-1}\right) & = & 1-\phi_{1}z^{-1}-\phi_{2}z^{-2}-\ldots -\phi_{p}z^{-p}\\ \Phi_{P}\left(z^{-s}\right) & = & 1-\Phi_{s}z^{-s}-\Phi_{2s}z^{-2s}-\ldots -\Phi_{Ps}z^{-Ps}\\ \theta_{q}\left(z^{-1}\right) & = & 1+\theta_{1}z^{-1}+\theta_{2}z^{-2}+\ldots +\theta_{q}z^{-q}\\ \Theta_{Q}\left(z^{-s}\right) & = & 1+\Theta_{s}z^{-s}+\Theta_{2s}z^{-2s}+\ldots +\Theta_{Qs}z^{-Qs}. \end{array}$$

Model (15) will be expressed in short form as SARIMA $\left(p,d,q\right){\left(P,D,Q\right)}_{s}$. If the clustering process is successful, the time subseries in a cluster will be similar, as defined by the partial distance measure *d*. Then, a SARIMA model can be identified for each cluster *i*, yielding a set of local glucose predictors (one per cluster). Remark that the cost index during identification will neglect blank data in the residuals computation, as well as pre-sampling data added during the concatenation. Box-Jenkins methodology [23] is used in this identification process. Exogenous inputs could also be considered with SARIMAX models [12], although here only glucose and mealtimes will be considered available.

**Figure 2:**
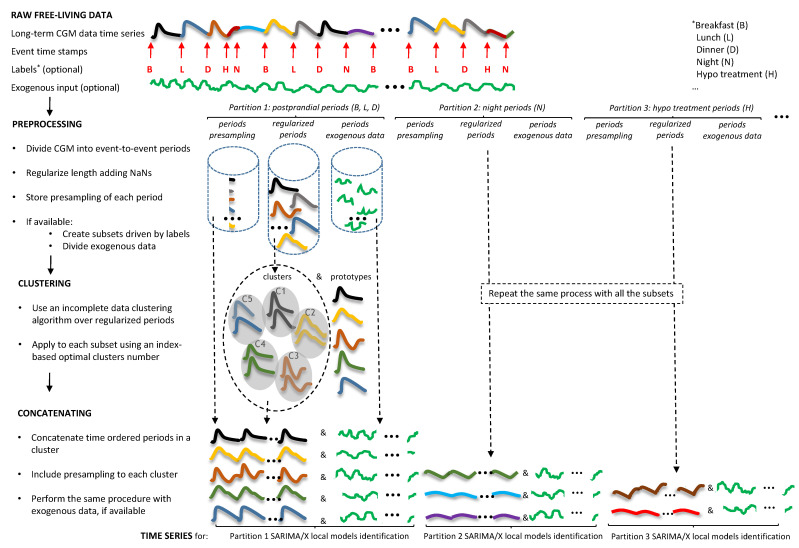
Data processing steps during the training phase. Optional event labels may include: Breakfast (B), Lunch (L), Dinner (D), Night (N), Hypo treatment (H), …

**Figure 3:**
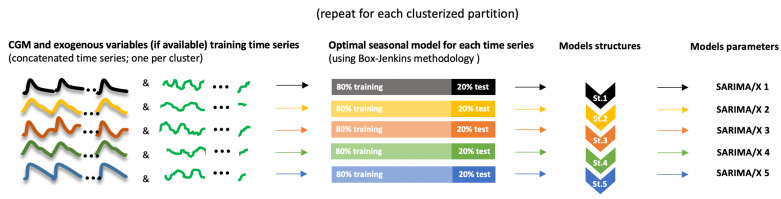
Local models identification process during the training phase. Data in a cluster was split in 80% for training and 20% for validation (see Section 2.7).

As a summary, Figure 2 shows the data processing steps during the training phase prior to local models identification. Figure 3 shows the identification procedure.

### 2.5. Model Integration

Once local models are trained for each cluster, a glucose predictor is built from the integration of such local models during real-time operation. Consider a given time $t_{p}\geq t_{e_{k}}$, where $t_{e_{k}}$ is the latest event timestamp, from which a prediction is to be computed according to the selected prediction horizon. A total of *c* local predictions will be available at that time instant, ${\hat{G}}_{i}\left(t|t_{p}\right)$, i=1,…,c produced by the models at each cluster. A global glucose prediction $\hat{G}\left(t|t_{p}\right)$ will be computed as a weighted sum of the *c* local predictions, with time-varying weights $\gamma_{i}\left(t_{p}\right)$, as follows

(17) $$\hat{G}\left(t|t_{p}\right):=\sum_{i=1}^{c}\gamma_{i}\left(t_{p}\right){\hat{G}}_{i}\left(t|t_{p}\right).$$

The computation of the weights $\gamma_{i}\left(t_{p}\right)$ is carried out in a two-step process in an attempt to discard coarsely non-contributing clusters (very dissimilar behavior of the cluster center compared to measured CGM data), and then refining the weighting among the remaining possibly contributing clusters. Given ${\left[G\right]}_{t_{0}}^{t_{1}}\left(t\right)$ the segment of CGM data from $t_{0}$ to $t_{1}$, and ${\left[\nu_{i}\right]}_{t_{0}}^{t_{1}}\left(t\right)$, i=1,…,c, the corresponding segments for cluster centers, the fuzzy membership of the segment ${\left[G\right]}_{t_{0}}^{t_{1}}\left(t\right)$ to the *i*-th cluster in a given index set *I* is defined as

(18) $$u_{i}\left(t_{1};t_{0}\right):={\left(\sum_{l=1}^{n_{I}}{\left(\frac{d^{2}\left({\left[G\right]}_{t_{0}}^{t_{1}}\left(t\right),{\left[\nu_{i}\right]}_{t_{0}}^{t_{1}}\left(t\right)\right)}{d^{2}\left({\left[G\right]}_{t_{0}}^{t_{1}}\left(t\right),{\left[\nu_{l}\right]}_{t_{0}}^{t_{1}}\left(t\right)\right)}\right)}^{\frac{1}{m-1}}\right)}^{-1},$$

where $n_{I}:=\left|I\right|$ is the cardinality of the index set *I*. Then, weights in (17) are computed as follows:

- First, fuzzy membership $u_{i}\left(t_{p};t_{e_{k}}\right)$, $i\in I_{1}:=\left\{1,\ldots ,c\right\}$ is computed. That is, similarity of CGM data and cluster centers from the event onset $t_{e_{k}}$ to current time $t_{p}$ is checked. Given a (possibly time-varying) threshold $\mu_{min}\left(t_{p}\right)$, a new index set $I_{2}:=\left\{i\in I_{1}\;|\;u_{i}\left(t_{p};t_{e_{k}}\right)\geq \mu_{min}\left(t_{p}\right)\right\}\subseteq I_{1}$ is defined, selecting the clusters with high enough similarity according to the selected $\mu_{min}$. A value $\mu_{min}\left(t_{p}\right):=0.2\max_{i}u_{i}\left(t_{p};t_{e_{k}}\right)$ was used here, that is, contributions of clusters for which membership is lower than 20% of the maximum membership are neglected.
- Then, in a second step, fuzzy membership $u_{i}\left(t_{p};t_{p}-W\right)$ to each cluster $i\in I_{2}$ is computed, where *W* is a to-be-defined (short) time window length. A time window *W* corresponding to 20 min will be considered here.

Weights $\gamma_{i}\left(t_{p}\right)$ are then computed as

(19) $$\gamma_{i}\left(t_{p}\right):=\left\{\begin{array}{cc} u_{i}\left(t_{p};t_{p}-W\right) & i\in I_{2}\\ 0 & i\in I_{1}\backslash I_{2}. \end{array}\right..$$

Remark that $\sum_{i=1}^{c}\gamma_{i}\left(t_{p}\right)=1$ and $\gamma_{i}\left(t_{p}\right)$ can be interpreted as a fuzzy membership value. We will refer to (17)–(19) as Global Seasonal Model (GSM) prediction.

It must be remarked that at the onset of the next event, $t_{e_{k+1}}$, the CGM time subseries from $t_{e_{k}}$ to $t_{e_{k+1}}$, with regularized length *L*, together with the corresponding pre-sampling data, must be appended as new data in the concatenated seasonal time series (Figure 1) of a selected cluster. This is needed because local SARIMA models make use of time-ordered previous data in the same cluster for their predictions, according to the SAR component order. The cluster with the highest fuzzy membership $u_{i}\left(t_{e_{k+1}};t_{e_{k}}\right)$, $i\in I_{1}$, for the CGM time subseries is selected for this purpose. This should not change representativeness of the cluster prototype, since high similarity of the new time subseries with respect to cluster members is expected, whenever training data represents well the diversity of glucose profiles in a patient. Eventually, online updating of the SARIMA models, or re-clustering and re-training, can be performed.

### 2.6. Real-Time Crispness and Normality Indices

Besides predicted glucose, two real-time indices are additionally provided indicating *crispness* of the prediction (degree of representation of current glucose behavior by a single cluster), and *normality* of the observed behavior, allowing for the detection of patient’s abnormal states (behaviors too dissimilar to available historical CGM data). It must be remarked that, in the scope of this work, the concept of normality is not primarily related to a physiological meaning: normality does not mean patient normality (normoglycemia), and abnormality does not mean patient abnormality (hyper- or hypoglycemia). On the contrary, abnormality must be understood as a behavior too dissimilar to available historical CGM data of a patient. For example, consider that patient A performed exercise regularly in the past and patient B did not perform exercise at all. This difference will be reflected in the available historical CGM data of patient A and B used during training, and, consequently, the identified models using data for patient A will be able to predict well a glycemic response under exercise, whereas identified models using data for patient B will perform badly when facing a well distinguishable response to exercise. Therefore, an exercise bout leading to the same CGM profile will be considered normal for patient A and abnormal for patient B. Hence, the objective of the normality index will be to inform patient B that something abnormal is happening in his/her CGM profile and that he/she can not rely on the prediction of the proposed system. In the case of patient A, nothing will happen in this case. In order to check the normality index capabilities and performance, in Section 2.1 a fictitious validation dataset 2 (“abnormal”) has been defined containing missed boluses and exercise sessions, which are not present in the validation dataset 1 (“normal”). Then, response to missed boluses and exercise sessions are expected to contain abnormal CGM segments (those well distinguishable from other glycemic responses in historical data) in the of the context of this paper, although missed boluses and exercise sessions are normal in the patients’ daily life. The crispness and normality indices may therefore provide significant information to the user (and control algorithms) as part of an online monitoring system.

The “crispness index” (CI) aims at providing information about how much the computed glucose prediction $\hat{G}\left(t|t_{p}\right)$ is produced by a single model. Since the glucose predictor is a multi-model system weighted by fuzzy membership values, the index rationale is that predictions based on a single local model in the set, perfectly matching the system’s behavior, should be more reliable. This ideal best case is the one with a membership value $\gamma_{i}\left(t_{p}\right)$ of 1 for one of the local models and 0 for the others. On the contrary, the worst case is that all local models contribute equally to the prediction, with weight 1/c. The crispness index at time $t_{p}$ is defined as the Manhattan distance, normalized to the interval [0, 1], between the vector composed by the membership values $\gamma_{i}\left(t_{p}\right)$, i=1,…,c, and the equally-distributed-membership vector (1/c,1/c,…,1/c), that is

(20) $$CI\left(t_{p}\right):=\frac{1}{2\left(1-\frac{1}{c}\right)}\sum_{i=1}^{c}\left|\gamma_{i}\left(t_{p}\right)-\frac{1}{c}\right|.$$

The “normality index” (NI) aims at determining the degree at which the current behavior is well represented in historical data and forecasting is the result of “interpolation” among local models predictions (normal behavior), or, on the contrary, the current behavior is beyond past behaviors and forecasting results from “extrapolation” by some or all the local models (abnormal behavior). Possibilistic memberships can give some hints in this case. The possibilistic counterpart of (18), denoted as $u_{i}^{P}\left(t_{1};t_{0}\right)$, representing possibilistic membership to the *i*-th cluster referred to the data window $\left[t_{0},t_{1}\right]$ can be computed as

(21) $$u_{i}^{P}\left(t_{1};t_{0}\right):={\left(1+\eta {\left(d^{2}\left({\left[G\right]}_{t_{0}}^{t_{1}}\left(t\right),{\left[\nu_{i}\right]}_{t_{0}}^{t_{1}}\left(t\right)\right)\right)}^{\frac{1}{m-1}}\right)}^{-1},$$

where $u_{i}^{P}\left(t_{1};t_{0}\right)\in \left[0,1\right]$, and η is a constant. Define now the possibilistic counterpart of (19) as

(22) $$\gamma_{i}^{P}\left(t_{p}\right):=\left\{\begin{array}{cc} u_{i}^{P}\left(t_{p};t_{p}-W\right) & i\in I_{2}\\ 0 & i\in I_{1}\backslash I_{2}. \end{array}\right.$$

Contrary to fuzzy membership, $\sum_{i=1}^{c}\gamma_{i}^{P}\left(t_{p}\right)=1$ does not necessarily hold now. In the presence of an abnormal period, far away and not represented by any cluster, equal memberships to each cluster would be obtained when considering fuzzy membership (18), resulting from similar distances to all prototypes. The same result would be obtained in a normal period just in the middle of the available clusters. Nevertheless, if the possibilistic membership (21) is used, such abnormal period would result in very low membership values for every cluster. Thus, at a given time $t_{p}$, the sum of all possibilistic memberships $\gamma_{i}^{P}\left(t_{p}\right)$ provides a measure of the sought concept: the closer to zero, the more abnormal the period is, not being represented enough by any cluster. Thus, the normality index is defined as

(23) $$NI\left(t_{p}\right):=\frac{1}{|I_{2}|}\sum_{i=1}^{c}\gamma_{i}^{P}\left(t_{p}\right),$$

where normalization by the number of non-zero membership values (cardinality of the index set $I_{2}$) is carried out so that $NI\left(t_{p}\right)\in \left[0,1\right]$. Abnormal behaviors may be due to hardware failures, extreme hypoglycemia or hyperglycemia, or any other glycemic behavior not represented in the historical time series used for training the local models. When abnormal behaviors are detected, the user should be informed of the extrapolation being performed. This can highlight the necessity of re-clustering and learning new local models. To this end, an abnormal behavior warning can be generated when $NI(t_{p})$ is below a given tunable threshold $NI_{thr}$.

**Figure 4:**
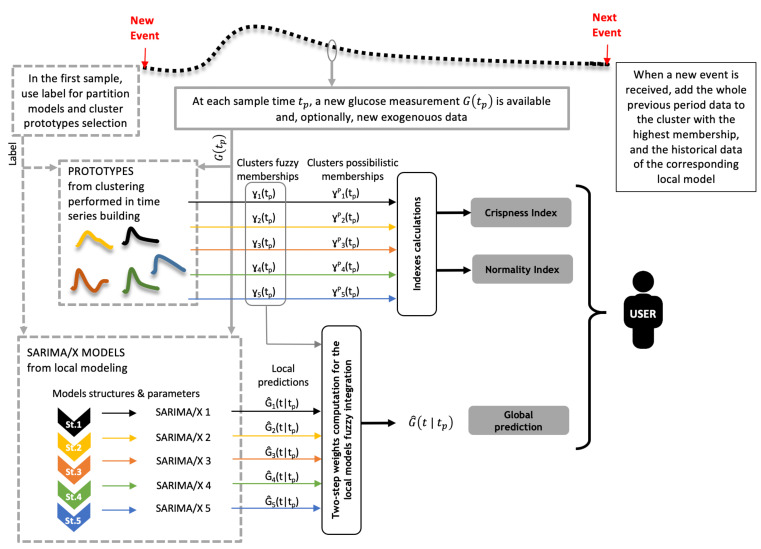
Real-time operation of the glucose predictor. When a new event is reported, the predictor trained for that event class (meal, night, or hypo treatment in this work) is selected. This predictor will be composed of a number of cluster prototypes and local models (dashed boxes PROTOTYPES and SARIMA/X MODELS). Then, at each sampling time $t_{p}$, a new glucose measurement $G(t_{p})$ is received from the CGM, and a predicted trajectory $\hat{G}\left(t|t_{p}\right)$ is computed considering a given prediction horizon. To this end, fuzzy memberships to each cluster, $\gamma_{i}\left(t_{p}\right)$, provide the weighting factors of predicted trajectories by the local models, ${\hat{G}}_{i}\left(t|t_{p}\right)$, following the two-step method described in Section 2.5. Additionally, crispness and normality indices are computed from fuzzy and possibilistic memberships to each cluster, respectively, as described in this section, which are provided as outputs together with the predicted trajectory. When the next event happens, the CGM time series of the last period is added to the cluster with most similar prototype, as well as to the historical data of the local model associated to that cluster.

Figure 4 summarizes the local models integration procedure to get a glucose prediction, together with the computation of crispness and normality indices.

### 2.7. Validation Procedure

The training data set comprised four-month in silico data for a ten patient cohort, as described in Section 2.1. For each patient, training data was partitioned in $\Pi_{M}$, $\Pi_{N}$, and $\Pi_{H}$ sets. Clustering and local model training was then applied to each partition, yielding a set of glucose predictors per patient (one per event group M, N, and H).

On the one hand, local model training was performed by separating data in each cluster into training and validation data. The first 80% of the available data for each cluster was used to generate the concatenated time series for model identification (Section 2.4). The remaining 20% was kept as validation time series. For all time subseries in the validation data in a cluster, predictions of the corresponding local model were evaluated, for prediction horizons PH∈{15,30,60,120,180,240} min, every time step, from starting time $t_{e_{i}}$ up to $t_{e_{i+1}}-PH$, in order to be able to compute prediction residuals. If the duration of the time subseries was smaller than PH, then it was discarded. Standard prediction horizons in the literature are 30 to 60 min. One of the purposes of this in silico study was to analyze in which extent these values could be extended, since longer prediction horizons can be beneficial, for instance, in the context of control algorithms. A prediction horizon of 240 min was considered long enough to represent a postprandial period, and longer prediction horizons were considered of no additional value. Standard metrics RMSE (mg/dL) and MAPE (%) were used to measure forecasting accuracy:

(24) $$RMSE:=\sqrt{\frac{1}{n}\sum_{i=1}^{n}e^{2}\left(i\right)}$$

(25) $$MAPE:=\frac{100}{n}\sum_{i=1}^{n}\left|\frac{e(i)}{G(i)},\right|$$

where *n* is the number of observations, G(i) denote the *i*th glucose observation, and $e\left(i\right)=G\left(i\right)-\hat{G}\left(i\right)$ is the forecasting error (residual), with $\hat{G}\left(i\right)$ the forecast of G(i). Two definitions of residuals were considered, leading to different forecasting accuracy metrics:

- Residuals of each predicted trajectory $G(t|t_{p})$, $t\in [t_{p},t_{p}+PH]$. It evaluates how successfully a predicted trajectory fits actual data. Remark that during the identification process glucose trajectories are sought to be fitted by the model, following the same rationale in this metrics definition. In this case, for each prediction, PH observations are available. Metrics were averaged for all computed predictions. Henceforth, this metrics will be referred to as $RMSE_{traj}$ and $MAPE_{traj}$. This is the metrics used in our previous works [11,12].
- Residuals of the glucose trajectory built from predictions $G(t_{p}+PH|t_{p})$. It evaluates how successfully actual glucose trajectory can be predicted PH time instants ahead. In this case, for each time subseries in the validation data, and denoting as $l_{i}$ the length of the time subseries, $l_{i}-PH$ observations are available for the computation of residuals. Metrics were averaged for all time subseries in the validation data. Henceforth, this metrics will be referred to as $RMSE_{last}$ and $MAPE_{last}$.

On the other hand, glucose predictors performance was evaluated using two independent 2-month validation data sets. A first validation data set included normal behavior, while the second one included new events not present in the training data in order to challenge the predictor (see Section 2.1). For each validation data set, glucose prediction was performed for the whole 2-month period, selecting the corresponding glucose predictor at each new event according to its type (meal, night, hypoglycemia treatment). For all time subseries in the validation data, predictions of the GSM model were computed in the same way as for local models evaluation, described above. As validation data was generated independently from training data, in few cases the duration of a validation time subseries was longer than the prototypes length (regularized length of training data). In this case, last value of the prototypes were held constant up to the validation time subseries length. Metrics $RMSE_{traj}$, $MAPE_{traj}$, $RMSE_{last}$, and $MAPE_{last}$ were computed as described before.

## 3. Results and Discussion

### 3.1. Time Series Building and Data Clustering

Training data, for each of the 10 patients, was comprised of 360 meals and 119 night events, of varying duration. The number of hypoglycemia treatment events was patient-dependent, attending to the settings of the open loop therapy provided in the simulator, with a median value of 18 events and 25–75% percentiles of 13 and 36 events, respectively. Mean CGM glucose ranged from 122 mg/dL (patient 10) to 169 mg/dL (patient 7).

Application of the PDSFCM clustering algorithm, in combination with the FS cluster validity index for the determination of the number of clusters, resulted in a varying number of clusters depending on the variability exhibited by the patient, as shown in Table 1.

**Figure 5:**
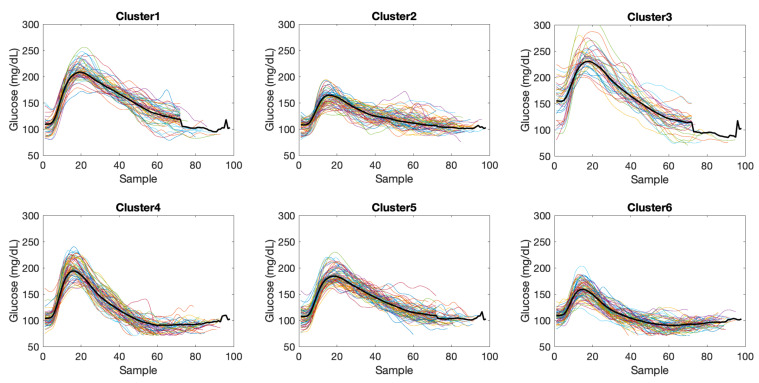
Clustering result of time subseries in meals partition $\Pi_{M}$ for patient 6. Black solid line indicates the cluster prototype.

As an illustration, Figure 5 shows the clustering results for patient 6 and data in the meals partition $\Pi_{M}$. The number of elements per cluster were 50, 61, 36, 63, 70 and 71 for clusters 1 to 6, respectively. Regularized length of the time subseries was 98. Considering the whole cohort, regularized lengths ranged from 98 to 106 samples for $\Pi_{M}$, from 60 to 65 samples for $\Pi_{N}$, and from 36 to 76 samples for $\Pi_{H}$.

After clustering, time subseries were assigned to the cluster with maximum membership. Then, a seasonal time series per cluster was created by concatenating all time subseries assigned to the given cluster representing similar glycemic excursions, preceded by pre-sampling data. A length of five samples was considered for pre-sampling data ($P_{r}=5$), given that orders lower than 5 were obtained for the AR and MA processes in previous studies. Thus, a seasonality period of s=L+5 was enforced, with *L* the regularized length for that patient and data partition. The resulting seasonalities are presented in Table 2.

### 3.2. Local Models Identification

A model for each cluster was identified using the Box-Jenkins methodology. As stated in Section 2.7, the first 80% of the data in a cluster were used for model identification, and the rest 20% for model evaluation. During the identification, residuals at time instants with missing values or belonging to pre-sampling periods in the concatenated time series were neglected (weighted 0 in the cost index). SARIMA model structure and parameters were identified for each cluster.

Table 3 shows, as an example, the resulting SARIMA model structure $\left(p,d,q\right){\left(P,D,Q\right)}_{s}$ for each cluster for patient 6. The orders for the AR component (*p*) ranges from 1 to 4, and for the MA component (*q*) from 0 to 4. With regards to seasonal part, SAR component order (*P*) ranges from 1 to 2, and SMA order (*Q*) from 0 to 2. Need for time series differentiation (d≠0 or D≠0) only appeared in hypoglycemia treatment cases, where glucose may follow an increasing trend. Similar results were obtained for the rest of patients.

Accuracy metrics of the trained local models is shown in Table 4, aggregating data for all patients and local models in a partition, as well as overall metrics. For all prediction horizons, the lowest metrics were obtained for the night partition $\Pi_{N}$ due to the lower variability during nights as compared to meals. Local models for $\Pi_{H}$ had a poorer performance due to the significantly lower number of events for training and validation, compared to $\Pi_{M}$ and $\Pi_{N}$. Comparing $RMSE_{traj}$$\left(MAPE_{traj}\right)$ versus $RMSE_{last}$$\left(MAPE_{last}\right)$, the latter reported higher values, since residuals of the last predicted value in each prediction is expected to be higher than when the whole predicted trajectory is considered. However, the higher the prediction horizon, the lower this difference was. This may be due to the implicit exclusion of residuals in $\left[t_{e_{i}},t_{e_{i}}+PH\right)$ in the computation of $RMSE_{last}$$\left(MAPE_{last}\right)$, where $t_{e_{i}}$ is the event initial time. When PH is long enough, an important part of a postprandial response may be neglected, which may provoke a loss of monotonicity of the $RMSE_{last}$$\left(MAPE_{last}\right)$ metrics with respect to the prediction horizon. Overall performance was good, with a maximum average $RMSE_{traj}$ of 11.46 mg/dL ($MAPE_{traj}$ of 6.79%), corresponding to a PH of 240 min. Maximum average $RMSE_{last}$ was 13.69 mg/dL (for a PH of 180 min), and maximum average $MAPE_{last}$ of 8.86% (for a PH of 240 min). During the night predictions, these metrics were reduced approximately by 40%.

### 3.3. Online Forecasting Validation

**Figure 6:**
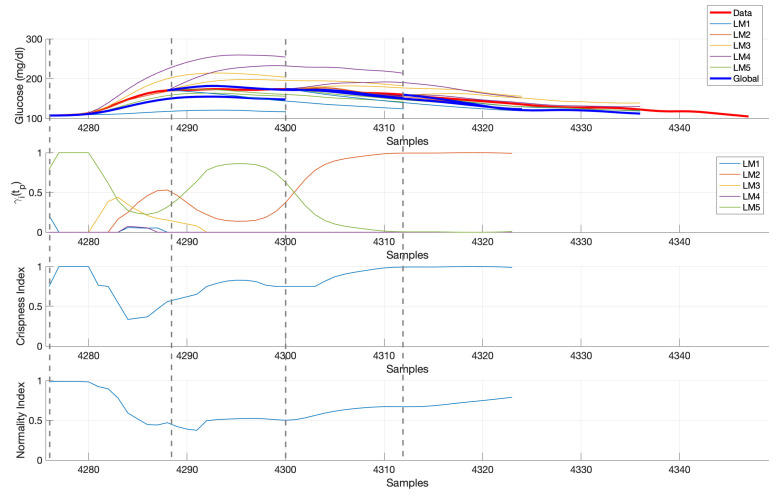
Illustration of the real-time computation of glucose predictions. Vertical dashed lines indicate the start of each 2-h prediction shown.

Figure 6 shows an illustration of the real-time computation of glucose predictions considering 5 local models for a sample validation time subseries in $\Pi_{M}$. Four 120-min ahead predictions are shown, separated one hour each, at the samples indicated by the vertical dashed lines starting at mealtime. Upper panel shows CGM data, local models predictions and global glucose prediction obtained from the weighted sum of local predictions, with time-varying weights are shown in the $\gamma_{i}\left(t_{p}\right)$ panel. Early postprandial response is more variable and, thus, more local models contribute to the glucose prediction computation, compared to late postprandial phase, where LM2 seems to fully describe the response in this case. This is also described by the crispness index, with lower values at time instants where a higher number of local models contribute to the predicted trajectory. The normality index describes to what extent past short-time CGM data at each prediction time $t_{p}$ is represented by the same segment of data from cluster prototypes, and “interpolation” instead of “extrapolation” is being performed. In this case, a drop of the index is observed during the glucose increase until peak value. A too low value would indicate an abnormal behavior, which threshold need to be tuned (see Section 3.4).

Table 5 present validation results of the glucose predictor in real-time operation for the 2-month independent validation dataset 1 (“normal”) described in Section 2.1. Metrics are expected to deteriorate as compared to the local models performance due to the local predictions integration process, as it is observed in the data presented. For this dataset, overall average $RMSE_{traj}$ ranged from 3.51 mg/dL to 23.90 mg/dL for prediction horizons 15, 30, 60, 120, 180 and 240 min ($MAPE_{traj}$ from 2.35% to 15.19%). Overall average $RMSE_{last}$ ranged from 6.52 mg/dL to 28.96 mg/dL ($MAPE_{last}$ from 4.04% to 20.00%).

Table 6 presents validation results for the two month independent validation dataset 2 (“abnormal”) described in Section 2.1. Inclusion of abnormal events slightly increased error in 1–2% as comparison of $MAPE_{traj}$ and $MAPE_{last}$ in Table 5 and Table 6 reveal. This was expected since missed boluses and exercise were not present in the training. The impact was higher up to a prediction horizon of 120 min, which may be related to the duration of the abnormal event effect on glycemia (postprandial and post-exercise periods). The number of predictions column indicates that abnormal events increased the number of hypoglycemic episodes (due to exercise), inducing different partitioning of data and participation of the glucose predictors for $\Pi_{M}$, $\Pi_{N}$, and $\Pi_{H}$. For the abnormal dataset, overall average $RMSE_{traj}$ ranged from 4.04 mg/dL to 25.36 mg/dL for the PHs considered ($MAPE_{traj}$ from 2.65% to 15.06%). Overall average $RMSE_{last}$ ranged from 7.33 mg/dL to 32.83 mg/dL ($MAPE_{last}$ from 4.66% to 19.82%).

Other authors have conducted in silico studies for the performance analysis of a variety of prediction methods, although comparison is not straightforward due to the difference in the simulators used, scenarios considered, and different input requirements by the glucose prediction technique. In this sense, the lower the inputs needed to perform a prediction the better, especially when such inputs require patient intervention such as the amount of carbs intake, for instance. These results are summarized in Table 7.

In [24], 8-day glucose and insulin data is used from a virtual cohort of 30 patients from the educational version of the UVA/Padova simulator, comprising 10 adults, 10 adolescents and 10 children. Variability in meal time and quantity was considered. Three models are compared: AR, ARX considering insulin as input, and ANN (artificial neural network), with prediction horizons of 30 min and 45 min. For the adult population, $RMSE_{last}$ values of 14.0 mg/dL, 13.3 mg/dL and 2.8 mg/dL are reported for AR, ARX and ANN with prediction horizon 30 min, respectively. These figures increase to 23.2 mg/dL, 22.8 mg/dL, and 4.0 mg/dL for a prediction horizon of 45 min. ANN outperformed AR and ARX models. Compared to results in Table 5, AR and ARX are outperformed by the methodology here presented, with a $RMSE_{last}$ of 11.32 mg/dL for a prediction horizon of 30 min. This is as well the case for results in Table 6 including abnormal data, where an $RMSE_{last}$ of 13.18 mg/dL was achieved. Metrics obtained for AR and ARX for 45 min are comparable to the metrics reported in Table 5 for a prediction horizon of 120 min ($RMSE_{last}=25.15$ mg/dL), and in Table 6 for 60 min ($RMSE_{last}=21.00$ mg/dL), respectively. However, this is not the case for ANN, with extremely low $RMSE_{last}$ values reported in [24]. This may be due to the nature of the in silico study, since the simulator used in [24] does not include the extra features of intra-patient variability reported in Section 2.1, besides the length of the data (8 day vs. 2 months), which might have produced overfitting in the ANN case. As well, the ANN used insulin data as external input which may limit applicability to insulin pump users, while in our case, only glucose and mealtime is used for glucose prediction. This latter can be extracted from the pump or smart pen information, or even estimated using meal detection algorithms in standard insulin pen users.

In [25], a neural network incorporating meal information in parallel with a linear predictor (NN-LPA) is evaluated in silico with 11-day data from 20 subjects of the UVA/Padova simulator. As in the previous case, variability in meal time and quantity was considered. The method is compared with the neural network presented in [30] (NNPG) and an AR(1) model. For a prediction horizon of 30 min, $RMSE_{last}$ values of 9.4 mg/dL, 10.7 mg/dL, and 17.5 mg/dL are reported for NN-LPA, NNPG and AR(1), respectively. Of note, NN-LPA requires a physiological meal model since the glucose rate of appearance at t+PH is one of the inputs of the neural network. In [26], a latent variable-based model (LVX) is evaluated from 7-day data from 10 virtual subjects of the UVA/Padova simulator. The model used meal intake and insulin information as exogenous variables. An average $RMSE_{last}$ of 8.6 mg/dL and 14.0 mg/dL was reported for 30-min and 60-min prediction horizons, respectively, as compared to our results in Table 5 with an $RMSE_{last}$ of 11.32 mg/dL and 17.85 mg/dL, respectively, and Table 6, with 13.18 mg/dL and 21.00 mg/dL, respectively. A slight increase of $RMSE_{last}$ of only approximately 3–4 mg/dL (5–7 mg/dL with abnormal data) is observed in our results, despite the far more challenging intra-patient variability in our work and less input information required, as stated above.

More recent in silico studies, in this case using the same extended UVA/Padova simulator as in this work, have been reported. In [27], a physiological model (PM) in combination with CGM signal deconvolution is presented for long-term glucose prediction. The model requires as inputs carbohydrate intake and insulin delivery, as opposed to our case. Two-week data for 10 in silico subjects is used for model training and evaluation. $RMSE_{last}$ values of 10.90 mg/dL, 24.44 mg/dL, 33.50 mg/dL, and 37.63 mg/dL are reported for prediction horizons of 30, 60, 90 and 120 min. These values outperformed ARX and LVX models in head-to-head comparisons. In [28], convolutional recurrent neural networks (CRNN) are evaluated with 1-year data from 10 in silico subjects. Models are trained with 50% of the data and evaluated with the rest 50%. Glucose, meal and insulin information are required as inputs. $RMSE_{last}$ values of 9.38 mg/dL and 18.87 mg/dL for 30-min and 60-min prediction horizons are reported. Above metrics are outperformed in general by results presented in Table 5 and Table 6, being especially relevant longer prediction horizons. Finally, in [29] dilated recurrent neural networks (DRNN) are tested with the same 1-year in silico study. A prediction horizon of 30 min is considered, with a reported $RMSE_{last}$ of 7.8 mg/dL, which is better than our result for the same prediction horizon even in the absence of abnormal data (11.32 mg/dL). However, DRNN required information on insulin, meal intake and time of the day, as opposed to this work.

Summing up, with the limitation of a non-head-to-head comparison and variety of simulation studies in the literature, metrics obtained outperformed other methods, or were very close to the reported performance. This was so even when imperfect training was considered introducing abnormal data in the validation dataset, while having the clear advantage of the minimal input information required (CGM and mealtime). This one can ultimately be automatically extracted without patient intervention making the glucose predictor suitable for insulin pump and MDI users.

From the point of view of the real-world application of the methodology, it is worth remarking that, in a similar way to a neural network, the training phase is the one more computationally demanding. In this work the computing cluster Rigel from Universitat Politècnica de València was used, in particular the Dell Power Edge R640 nodes with 2 processors Intel Xeon Gold 6154 with 18 cores, 3 Ghz and 25 Mb cache memory. The clustering phase for the 10 patients, which included 10 clustering problems per patient with increasing number of clusters for later selecting the optimal number of them, using 22 cores and 3 Gb memory per core, was computed in 22 min. The training of the local models, using 30 cores and 3 Gb memory per core, was performed in about 6.5 h. Validation of local models, using the same resources, lasted 53 min. This gives rise to a total of 8 h of computing time approximately for training. Once trained, the real-time evaluation of a glucose prediction is straightforward, requiring the evaluation of equations in Section 2.5 for the glucose prediction (evaluation of *n* SARIMA models, *n* fuzzy memberships, and a weighted sum of time series, where *n* is the number of clusters), and Section 2.6 for crispness and normality indices.

### 3.4. Normality Index

**Figure 7:**
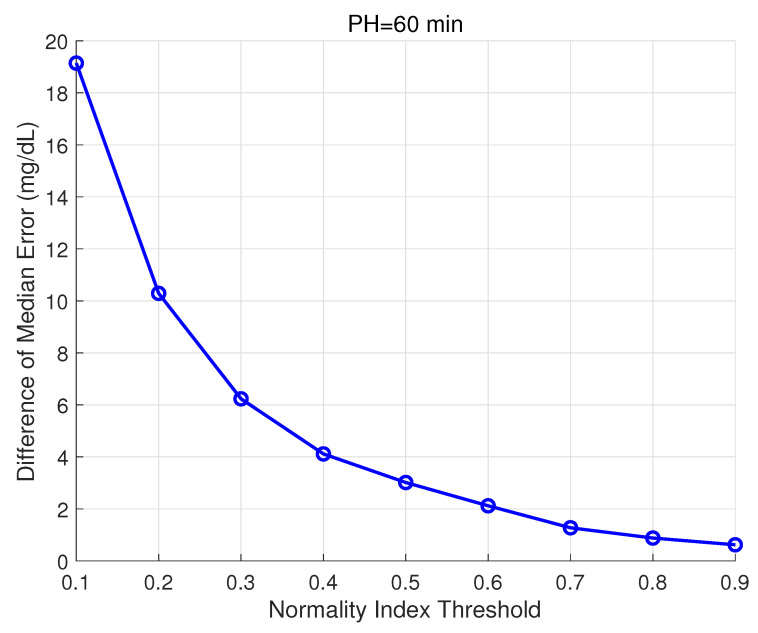
Relationship between Normality Index and prediction error. Difference of the median absolute value of the residual at $t_{p}+PH$ between groups for $NI\left(t_{p}\right)<NI_{thr}$ and $NI\left(t_{p}\right)\geq NI_{thr}$, with $NI_{thr}$ ranging from 0.1 to 0.9, is shown. All differences were found statistically significant (p<0.001).

The Normality Index defined in Equation (23) is intended to provide information on abnormal glucose behaviors according to available historical data. Thus, it is expected that low normality values at time $t_{p}$, $NI(t_{p})$, are related to higher prediction errors for glucose trajectories computed at that same time $t_{p}$. The Normality Index relies on the comparison of CGM values in the recent past (20 min in this work) with cluster prototypes in that same time window. It is expected that this relationship with the prediction error is stronger for short or medium prediction horizons, for which abnormal behavior is still present. A PH of 60 min was considered in the following analysis. All predictions $G(t|t_{p})$, $t\in [t_{p},t_{p}+PH]$, carried out during the validation of the glucose predictor for dataset 2 (“abnormal”), amounting to a total of 138,888 predicted trajectories, were grouped into two groups, for $NI\left(t_{p}\right)<NI_{thr}$ and $NI\left(t_{p}\right)\geq NI_{thr}$, with the threshold $NI_{thr}$ varying from 0.1 to 0.9 in steps of 0.1. Then, the difference of the median prediction error at $t_{p}+PH$ (absolute value of the residual) among groups was analyzed. Remark that these residuals are the ones considered in the computation of $RMSE_{last}$ for a given validation time subseries. Figure 7 shows these results. All differences were found statistically significant using the Wilcoxon Rank Sum test (p<0.001). This test was selected since data was non-normal and non-paired.

**Figure 8:**
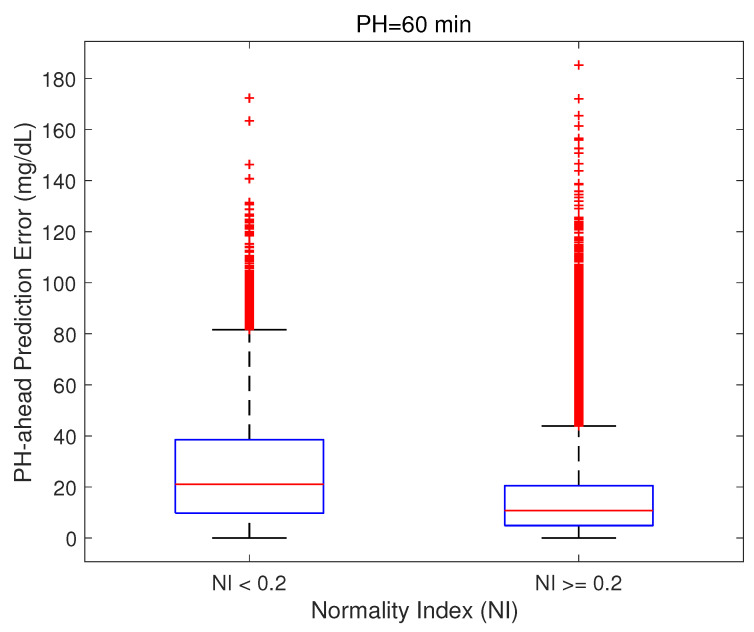
Boxplot of absolute value of the residual at $t_{p}+PH$ grouped by Normality Index value for a threshold of 0.2. Difference of the median was statistically significant by Wilcoxon Rank Sum test (p<0.001).

Clinically meaningful differences were obtained for the lowest thresholds, amounting to 19.14 mg/dL for $NI_{thr}=0.1$, and 10.29 mg/dL for $NI_{thr}=0.2$. As an illustration, Figure 8 shows the relationship for $NI_{thr}=0.2$, which reveals an increased presence of higher prediction errors when $NI(t_{p})<0.2$ (median [25th–75th percentiles]: 21.05 [9.79, 38.52] vs. 10.75 [4.88, 20.49] mg/dL).

**Figure 9:**
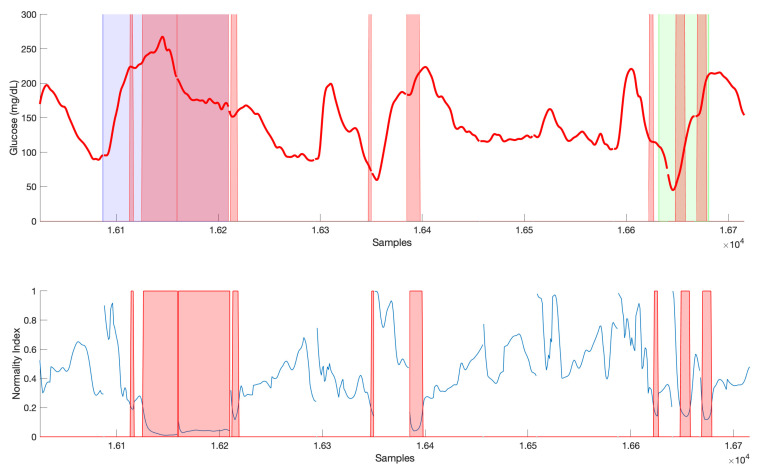
Example of relationship between Normality Index and abnormal events in the simulated scenario. Shaded blue area indicates a missed bolus postprandial period. Shaded green area indicates an exercise session and 3-h post-exercise period. Shaded red area indicates a section of CGM data classified as abnormal ($NI(t_{p})<0.2$).

Finally, Figure 9 illustrates an example of the relationship between NI and abnormal events in the simulated scenario. Postprandial response after a missed bolus (blue shaded area) is clearly detected as an abnormal CGM response. Consider that a minimum delay of 20 min will happen since that is the window considered in the computation of NI. After an initial glucose rise that could be similar to other meals, the value of NI is below the threshold consecutively at each sample during the postprandial period until glucose returns to normoglycemia. Regarding the exercise, including 3-h post-exercise period with altered insulin sensitivity (green area), abnormality is found in the hypoglycemia recovery, due to the increased need of carbohydrates as compared to a non-exercise related hypoglycemia in the training data, as well as the initial response of the meal following exercise. Other segments of CGM data might be also considered abnormal without relation to missed boluses or exercise, such as the one around sample $1.64\times 10^{4}$. In this case, a meal close to a hypoglycemia recovery happened.

The information provided by the Normality Index can be useful in real-time operation, raising for instance warnings when sufficiently long abnormal periods are detected (as in the case of missed boluses), due to expected decrease of accuracy of predictions. Additionally, remark that its computation is independent of predictions since it relies only on the clustering training phase (no local models are implied). This means that its use in an offline context can also be devised, as a tool for the analysis of CGM data highlighting areas of abnormal response that may deserve special attention by the clinician.

## 4. Conclusions

In previous works, seasonal local modeling proved successful in glucose prediction for longer prediction horizons. However, fixed-length postprandial time series were used, which do not apply to realistic scenario. In this work, this limitation is overcome with the introduction of event-to-event CGM time series partitioning, and clustering of variable-length data. A new local models integration method is also proposed. Metrics obtained outperformed other literature methods, or were very close to the reported ones, even when imperfect training was considered introducing abnormal data in the validation dataset, while having the clear advantage of the minimal input information required (CGM and mealtime). This one can ultimately be automatically extracted without patient intervention making the glucose predictor suitable for insulin pump and MDI users. Besides, the normality index can provide useful information about abnormal glycemic responses leading to a more cautious use of the provided predictions, or providing valuable information to clinicians for the inspection of CGM data. This study has the limitation of any in silico study, and evaluation with clinical data should be conducted as the next step in this research. Although this work focused on the adult cohort for the sake of comparison with other literature methods, the methodology could have been applied equally to the adolescent or children virtual cohort, with an adaptation of the number of clusters considered according to the population variability.

## Figures and Tables

**Table 1:** Number of clusters considered for each patient (P) according to the FS cluster validity index. For patients 5 and 8, not enough hypoglycemia treatments were available to apply clustering (marked as *). In these cases, only a single local model is considered.

	P1	P2	P3	P4	P5	P6	P7	P8	P9	P10
**Meals ($\Pi_{M}$)**	5	5	5	5	6	6	8	5	5	5
**Night ($\Pi_{N}$)**	3	3	3	3	3	3	4	3	4	5
**Hypo treatment ($\Pi_{H}$)**	2	2	2	2	1 *	3	4	1 *	3	3

**Table 2:** Seasonality (in number of samples) resulting from time subseries concatenation in a cluster.

	P1	P2	P3	P4	P5	P6	P7	P8	P9	P10
**Meals ($\Pi_{M}$)**	111	105	106	103	105	103	103	107	103	105
**Night ($\Pi_{N}$)**	70	66	65	74	67	68	69	66	66	68
**Hypo treatment ($\Pi_{H}$)**	81	51	60	75	43	74	65	41	65	61

**Table 3:** Example of local models structure (patient 6), represented as $\left(p,d,q\right){\left(P,D,Q\right)}_{s}$.

Partition	Cluster	SARIMA Model Structure
Meals ($\Pi_{M}$)	1	$\left(2,0,1\right){\left(2,0,2\right)}_{103}$
	2	$\left(2,0,2\right){\left(1,0,1\right)}_{103}$
	3	$\left(4,0,4\right){\left(2,0,0\right)}_{103}$
	4	$\left(3,0,3\right){\left(2,0,1\right)}_{103}$
	5	$\left(4,0,1\right){\left(1,0,1\right)}_{103}$
	6	$\left(3,0,2\right){\left(1,0,1\right)}_{103}$
Night ($\Pi_{N}$)	1	$\left(2,0,0\right){\left(1,0,2\right)}_{68}$
	2	$\left(2,0,4\right){\left(1,0,1\right)}_{68}$
	3	$\left(2,0,3\right){\left(1,0,1\right)}_{68}$
Hypo treatment ($\Pi_{H}$)	1	$\left(4,0,0\right){\left(1,0,1\right)}_{74}$
	2	$\left(1,0,2\right){\left(1,0,1\right)}_{74}$
	3	$\left(1,1,0\right){\left(1,1,0\right)}_{74}$

**Table 4:** Accuracy metrics of the trained local models aggregating data for all patients and local models in a partition, as well as overall metrics. The number of computed PH-ahead predictions is reported in each case, which depends on the number of validation events and their length.

	Number of	${\mathrm{RMSE}}_{\mathrm{traj}}$	${\mathrm{RMSE}}_{\mathrm{last}}$	${\mathrm{MAPE}}_{\mathrm{traj}}$	${\mathrm{MAPE}}_{\mathrm{last}}$
	Predictions	(mg/dL)	(mg/dL)	(%)	(%)
**PH = 15 min**					
**Meals ($\Pi_{M}$)**	54,365	3.03 (2.46)	5.46 (1.11)	1.96 (1.61)	2.89 (0.43)
		2.41 [1.22, 4.16]	5.24 [4.79, 5.95]	1.53 [0.76, 2.70]	2.91 [2.58, 3.23]
**Night ($\Pi_{N}$)**	10,940	2.34 (1.98)	4.60 (2.54)	1.91 (1.56)	3.04 (1.38)
		1.84 [0.97, 3.15]	3.94 [3.55, 4.32]	1.51 [0.76, 2.60]	2.84 [2.43, 3.23]
**Hypo treatment ($\Pi_{H}$)**	1440	3.98 (3.60)	6.71 (2.78)	3.17 (3.19)	4.78 (2.30)
		3.02 [1.46, 5.21]	5.79 [4.80, 7.69]	2.16 [1.09, 4.03]	4.12 [3.11, 5.90]
**Overall**	66,745	2.94 (2.43)	5.45 (2.15)	1.97 (1.66)	3.32 (1.50)
		2.31 [1.17, 4.00]	5.01 [4.16, 6.03]	1.54 [0.77, 2.70]	3.04 [2.59, 3.33]
**PH = 30 min**					
**Meals ($\Pi_{M}$)**	52,187	4.98 (3.76)	9.10 (1.94)	3.09 (2.36)	4.80 (0.76)
		4.01 [2.26, 6.68]	8.53 [7.99, 9.84]	2.46 [1.35, 4.17]	4.80 [4.19, 5.40]
**Night ($\Pi_{N}$)**	10,211	3.64 (2.86)	7.09 (4.49)	2.87 (2.17)	4.67 (2.49)
		2.92 [1.74, 4.73]	5.87 [5.27, 6.99]	2.29 [1.33, 3.78]	4.24 [3.75, 4.80]
**Hypo treatment ($\Pi_{H}$)**	1304	6.71 (6.01)	11.78 (6.11)	4.92 (4.66)	7.95 (5.10)
		4.88 [2.70, 8.57]	10.44 [7.64, 13.57]	3.32 [1.81, 6.45]	6.85 [4.74, 8.67]
**Overall**	63,702	4.80 (3.74)	9.04 (4.24)	3.09 (2.41)	5.41 (3.00)
		3.80 [2.16, 6.39]	8.08 [6.59, 10.30]	2.45 [1.36, 4.13]	4.75 [4.06, 5.52]
**PH = 60 min**					
**Meals ($\Pi_{M}$)**	47,831	7.45 (5.17)	12.52 (3.19)	4.50 (3.13)	6.68 (1.21)
		6.13 [3.77, 9.69]	11.69 [10.63, 13.92]	3.70 [2.22, 5.95]	6.63 [5.67, 7.52]
**Night ($\Pi_{N}$)**	8753	4.94 (3.58)	8.86 (6.82)	3.80 (2.59)	5.79 (3.86)
		4.10 [2.67, 6.11]	6.84 [6.07, 8.26]	3.15 [2.00, 4.87]	4.92 [4.22, 6.03]
**Hypo treatment ($\Pi_{H}$)**	1034	10.53 (9.53)	17.23 (12.39)	7.04 (6.49)	10.67 (9.46)
		7.31 [4.21, 13.55]	14.42 [9.22, 21.76]	4.59 [2.75, 8.95]	6.77 [4.84, 14.34]
**Overall**	57,618	7.13 (5.17)	12.37 (7.61)	4.44 (3.18)	7.23 (5.11)
		5.73 [3.54, 9.18]	10.77 [7.85, 14.01]	3.62 [2.18, 5.81]	6.09 [4.98, 7.50]
**PH = 120 min**					
**Meals ($\Pi_{M}$)**	39,119	9.82 (6.09)	14.19 (4.37)	5.88 (3.74)	8.10 (1.72)
		8.32 [5.57, 12.39]	13.29 [11.52, 15.35]	4.98 [3.22, 7.54]	7.95 [6.81, 9.13]
**Night ($\Pi_{N}$)**	5837	5.98 (3.85)	9.41 (8.48)	4.47 (2.58)	6.19 (5.17)
		5.26 [3.75, 7.21]	7.15 [6.16, 9.10]	3.86 [2.75, 5.50]	5.15 [4.30, 6.67]
**Hypo treatment ($\Pi_{H}$)**	522	16.63 (14.44)	19.27 (13.96)	10.36 (9.23)	10.50 (7.85)
		10.54 [6.32, 21.66]	15.53 [8.60, 27.97]	6.54 [3.80, 13.45]	7.56 [4.36, 15.76]
**Overall**	45,478	9.40 (6.20)	13.58 (8.72)	5.75 (3.78)	7.92 (4.72)
		7.79 [5.21, 11.81]	12.00 [8.12, 15.26]	4.82 [3.14, 7.28]	7.16 [5.34, 8.84]
**PH = 180 min**					
**Meals ($\Pi_{M}$)**	30,413	10.89 (6.09)	14.16 (4.75)	6.49 (3.77)	8.58 (1.97)
		9.50 [6.63, 13.52]	13.05 [11.02, 15.40]	5.57 [3.83, 8.22]	8.77 [6.99, 9.80]
**Night ($\Pi_{N}$)**	2935	6.45 (3.67)	9.96 (9.64)	4.78 (2.42)	6.76 (6.35)
		5.84 [4.32, 7.55]	7.59 [6.07, 9.85]	4.21 [3.12, 5.91]	5.12 [4.28, 7.63]
**Hypo treatment ($\Pi_{H}$)**	193	20.73 (17.21)	21.48 (16.47)	12.49 (10.47)	14.37 (10.50)
		12.87 [7.47, 30.05]	13.46 [11.33, 32.33]	6.95 [4.66, 20.11]	11.06 [5.31, 22.06]
**Overall**	33,541	10.56 (6.22)	13.69 (9.34)	6.37 (3.80)	8.71 (5.81)
		9.06 [6.30, 13.11]	11.67 [9.31, 14.71]	5.45 [3.74, 8.01]	7.67 [5.65, 9.57]
**PH = 240 min**					
**Meals ($\Pi_{M}$)**	21,865	11.56 (5.88)	14.32 (4.89)	6.81 (3.54)	9.06 (2.21)
		10.39 [7.45, 14.13]	13.21 [10.68, 15.99]	6.00 [4.27, 8.52]	8.97 [7.09, 10.81]
**Night ($\Pi_{N}$)**	576	6.71 (2.50)	8.34 (4.44)	5.12 (2.13)	6.37 (4.14)
		6.30 [4.77, 8.25]	7.71 [4.99, 9.29]	4.87 [3.53, 6.14]	4.96 [3.62, 7.08]
**Hypo treatment ($\Pi_{H}$)**	56	23.53 (17.21)	27.74 (29.20)	13.94 (10.27)	19.55 (20.59)
		16.13 [9.71, 34.75]	13.24 [9.86, 51.20]	8.51 [5.36, 21.68]	10.09 [4.05, 43.91]
**Overall**	22,497	11.46 (5.95)	13.23 (9.53)	6.79 (3.58)	8.86 (6.46)
		10.27 [7.32, 14.02]	11.72 [8.68, 15.08]	5.96 [4.25, 8.47]	7.93 [5.82, 10.81]

**Table 5:** Accuracy metrics of the glucose predictor for validation dataset 1 (“normal”).

	Number of	${\mathrm{RMSE}}_{\mathrm{traj}}$	${\mathrm{RMSE}}_{\mathrm{last}}$	${\mathrm{MAPE}}_{\mathrm{traj}}$	${\mathrm{MAPE}}_{\mathrm{last}}$
	Predictions	(mg/dL)	(mg/dL)	(%)	(%)
**PH = 15 min**					
**Meals ($\Pi_{M}$)**	133,540	3.63 (3.11)	6.58 (0.96)	2.36 (2.00)	3.55 (0.36)
		2.81 [1.39, 4.93]	6.54 [6.10, 6.79]	1.83 [0.90, 3.22]	3.56 [3.34, 3.85]
**Night ($\Pi_{N}$)**	26,426	2.79 (2.31)	4.90 (0.87)	2.14 (1.75)	3.17 (0.63)
		2.19 [1.13, 3.80]	4.71 [4.29, 5.07]	1.67 [0.84, 2.96]	3.00 [2.73, 3.69]
**Hypo treatment ($\Pi_{H}$)**	4308	3.99 (3.35)	8.08 (2.97)	3.46 (3.15)	5.41 (1.68)
		3.18 [1.59, 5.42]	6.57 [6.43, 10.80]	2.57 [1.25, 4.68]	5.10 [4.46, 6.15]
**Overall**	164,274	3.51 (3.02)	6.52 (2.24)	2.35 (2.01)	4.04 (1.42)
		2.71 [1.35, 4.75]	6.37 [5.07, 6.70]	1.82 [0.89, 3.21]	3.63 [3.09, 4.46]
**PH = 30 min**					
**Meals ($\Pi_{M}$)**	128,144	6.31 (5.26)	12.08 (1.81)	3.90 (3.17)	6.35 (0.67)
		4.88 [2.61, 8.44]	11.71 [11.16, 12.33]	3.05 [1.61, 5.26]	6.30 [5.84, 6.87]
**Night ($\Pi_{N}$)**	24,659	4.50 (3.57)	7.93 (1.83)	3.34 (2.63)	5.09 (1.16)
		3.58 [2.04, 5.90]	7.42 [6.69, 8.35]	2.64 [1.49, 4.46]	4.63 [4.39, 5.79]
**Hypo treatment ($\Pi_{H}$)**	3746	6.96 (5.37)	13.95 (4.67)	5.60 (4.82)	9.05 (3.48)
		5.49 [3.12, 9.35]	12.66 [11.30, 14.53]	4.15 [2.25, 7.50]	7.90 [7.35, 9.95]
**Overall**	156,549	6.04 (5.08)	11.32 (3.92)	3.86 (3.16)	6.83 (2.67)
		4.64 [2.51, 8.03]	11.27 [8.35, 12.65]	3.00 [1.60, 5.16]	6.47 [5.55, 7.37]
**PH = 60 min**					
**Meals ($\Pi_{M}$)**	117,356	10.45 (8.60)	19.49 (3.52)	6.30 (4.97)	10.19 (1.42)
		8.06 [4.54, 13.65]	18.58 [17.43, 20.09]	4.93 [2.75, 8.34]	9.76 [9.28, 10.76]
**Night ($\Pi_{N}$)**	21,142	6.50 (4.98)	10.46 (2.95)	4.74 (3.57)	6.63 (1.73)
		5.22 [3.33, 8.16]	9.67 [8.23, 11.08]	3.79 [2.35, 6.05]	6.16 [5.58, 7.18]
**Hypo treatment ($\Pi_{H}$)**	2709	12.25 (9.24)	23.60 (9.77)	8.87 (7.20)	13.75 (6.65)
		9.59 [5.42, 16.40]	20.94 [17.57, 23.39]	6.62 [3.58, 12.05]	11.07 [10.60, 15.28]
**Overall**	141,207	9.90 (8.30)	17.85 (8.21)	6.11 (4.89)	10.19 (4.90)
		7.48 [4.29, 12.83]	17.44 [11.08, 20.93]	4.74 [2.69, 8.04]	9.68 [7.18, 10.76]
**PH = 120 min**					
**Meals ($\Pi_{M}$)**	95,780	16.30 (12.49)	26.80 (6.03)	10.03 (7.72)	15.87 (3.10)
		12.86 [7.65, 21.14]	24.77 [22.77, 28.19]	7.87 [4.61, 13.09]	14.99 [13.52, 17.46]
**Night ($\Pi_{N}$)**	14,149	8.61 (6.18)	12.01 (3.62)	6.23 (4.48)	7.91 (2.50)
		7.08 [4.78, 10.50]	11.24 [8.71, 14.29]	5.05 [3.33, 7.76]	7.52 [5.83, 9.14]
**Hypo treatment ($\Pi_{H}$)**	1143	21.67 (17.55)	36.64 (21.12)	13.85 (10.65)	20.00 (10.06)
		15.65 [9.02, 28.52]	29.97 [20.72, 42.83]	9.97 [5.51, 19.77]	16.31 [14.01, 22.75]
**Overall**	111,072	15.38 (12.23)	25.15 (16.12)	9.58 (7.55)	14.59 (7.90)
		11.80 [7.02, 19.90]	22.72 [14.29, 28.19]	7.40 [4.36, 12.41]	13.77 [9.14, 16.67]
**PH = 180 min**					
**Meals ($\Pi_{M}$)**	74,297	21.02 (14.45)	32.14 (6.80)	13.36 (9.96)	21.57 (4.41)
		17.24 [10.62, 27.63]	30.31 [27.52, 34.38]	10.49 [6.35, 17.48]	21.58 [17.81, 22.70]
**Night ($\Pi_{N}$)**	7182	10.71 (6.55)	13.25 (4.41)	7.71 (4.74)	9.21 (3.03)
		9.18 [6.37, 13.01]	13.32 [9.33, 14.79]	6.56 [4.41, 9.48]	9.35 [6.50, 10.43]
**Hypo treatment ($\Pi_{H}$)**	374	26.67 (20.46)	41.50 (25.48)	16.83 (12.91)	27.95 (16.87)
		20.94 [10.72, 34.96]	35.28 [29.39, 41.73]	12.15 [6.28, 25.29]	20.88 [16.53, 34.55]
**Overall**	81,853	20.15 (14.28)	28.96 (19.10)	12.88 (9.77)	19.58 (12.64)
		16.24 [9.92, 26.56]	28.45 [14.79, 34.38]	10.00 [6.07, 16.78]	17.74 [9.73, 22.01]
**PH = 240 min**					
**Meals ($\Pi_{M}$)**	53,118	24.19 (16.07)	34.53 (9.29)	15.36 (10.99)	24.21 (6.91)
		20.05 [12.60, 31.81]	33.93 [27.40, 37.77]	12.36 [7.63, 19.91]	23.35 [21.10, 27.92]
**Night ($\Pi_{N}$)**	1384	11.83 (6.66)	12.87 (6.05)	8.31 (4.90)	9.04 (4.09)
		9.59 [7.56, 14.32]	10.38 [8.30, 16.19]	6.85 [5.13, 10.19]	7.54 [5.85, 12.28]
**Hypo treatment ($\Pi_{H}$)**	117	32.68 (20.11)	41.58 (14.18)	21.51 (14.59)	33.52 (26.41)
		26.69 [16.15, 48.10]	43.77 [30.66, 50.34]	18.86 [9.74, 33.57]	23.21 [16.02, 45.16]
**Overall**	54,619	23.90 (16.04)	27.28 (15.18)	15.19 (10.94)	20.00 (15.36)
		19.70 [12.28, 31.40]	25.58 [13.10, 36.60]	12.17 [7.50, 19.71]	17.04 [9.15, 24.53]

**Table 6:** Accuracy metrics of the glucose predictor for validation dataset 2 (“abnormal”).

	Number of	${\mathrm{RMSE}}_{\mathrm{traj}}$	${\mathrm{RMSE}}_{\mathrm{last}}$	${\mathrm{MAPE}}_{\mathrm{traj}}$	${\mathrm{MAPE}}_{\mathrm{last}}$
	Predictions				
**PH = 15 min**					
**Meals ($\Pi_{M}$)**	128,002	4.18 (3.72)	7.72 (1.12)	2.59 (2.34)	3.92 (0.34)
		3.12 [1.52, 5.63]	7.51 [7.17, 8.18]	1.96 [0.95, 3.50]	3.95 [3.72, 4.25]
**Night ($\Pi_{N}$)**	26,113	3.14 (2.86)	5.70 (1.17)	2.25 (1.80)	3.33 (0.52)
		2.35 [1.20, 4.15]	5.68 [5.17, 6.08]	1.79 [0.88, 3.17]	3.34 [2.90, 3.64]
**Hypo treatment ($\Pi_{H}$)**	9496	4.71 (4.57)	8.57 (2.80)	4.58 (4.69)	6.74 (1.57)
		3.64 [1.75, 6.40]	7.27 [6.04, 11.59]	3.09 [1.41, 6.16]	6.69 [5.48, 7.72]
**Overall**	163,611	4.04 (3.68)	7.33 (2.18)	2.65 (2.51)	4.66 (1.78)
		3.00 [1.47, 5.44]	6.99 [5.97, 8.18]	1.97 [0.95, 3.55]	4.08 [3.43, 5.48]
**PH = 30 min**					
**Meals ($\Pi_{M}$)**	122,617	7.29 (6.34)	14.34 (2.13)	4.31 (3.73)	7.13 (0.59)
		5.42 [2.84, 9.65]	13.69 [13.14, 15.10]	3.29 [1.70, 5.75]	7.04 [6.55, 7.72]
**Night ($\Pi_{N}$)**	24,349	5.06 (4.45)	9.35 (2.10)	3.53 (2.66)	5.38 (0.95)
		3.79 [2.16, 6.54]	9.33 [8.42, 9.87]	2.79 [1.54, 4.86]	5.31 [4.69, 5.75]
**Hypo treatment ($\Pi_{H}$)**	8374	8.40 (7.17)	15.84 (4.89)	7.53 (7.11)	11.52 (2.10)
		6.74 [3.55, 11.33]	14.03 [11.80, 20.75]	5.39 [2.66, 10.05]	11.66 [9.82, 12.43]
**Overall**	155,340	7.00 (6.20)	13.18 (4.26)	4.36 (3.93)	8.01 (2.94)
		5.15 [2.71, 9.24]	12.70 [9.87, 15.10]	3.27 [1.70, 5.77]	7.05 [5.75, 9.82]
**PH = 60 min**					
**Meals ($\Pi_{M}$)**	111,847	12.16 (10.46)	23.62 (4.20)	6.99 (5.82)	11.73 (1.20)
		8.92 [4.98, 15.79]	22.89 [20.89, 24.46]	5.33 [2.92, 9.24]	11.29 [10.76, 12.60]
**Night ($\Pi_{N}$)**	20,823	7.35 (6.33)	12.65 (3.58)	5.04 (3.56)	7.10 (1.36)
		5.52 [3.42, 9.04]	12.60 [11.17, 13.81]	4.04 [2.40, 6.74]	7.14 [6.48, 7.67]
**Hypo treatment ($\Pi_{H}$)**	6218	14.75 (11.19)	26.73 (9.93)	11.76 (10.03)	15.87 (4.64)
		11.70 [6.66, 19.95]	25.76 [17.93, 33.63]	8.79 [4.56, 16.33]	14.84 [12.01, 19.65]
**Overall**	138,888	11.56 (10.16)	21.00 (8.82)	6.91 (5.94)	11.57 (4.58)
		8.34 [4.66, 14.92]	20.04 [13.81, 24.46]	5.17 [2.86, 9.05]	10.86 [7.67, 12.98]
**PH = 120 min**					
**Meals ($\Pi_{M}$)**	90,307	19.04 (15.74)	33.75 (7.58)	11.13 (9.08)	18.56 (2.67)
		14.30 [8.20, 24.39]	31.51 [30.93, 36.49]	8.57 [4.77, 14.67]	17.71 [17.02, 19.02]
**Night ($\Pi_{N}$)**	13,795	9.90 (8.78)	15.26 (5.67)	6.65 (4.54)	8.44 (2.12)
		7.26 [4.79, 11.98]	13.59 [11.92, 18.08]	5.20 [3.42, 8.70]	8.48 [7.29, 10.16]
**Hypo treatment ($\Pi_{H}$)**	2536	25.96 (19.05)	38.90 (19.18)	17.53 (12.18)	20.16 (9.07)
		20.77 [12.62, 33.16]	40.80 [18.61, 51.14]	14.87 [8.38, 23.88]	21.59 [9.67, 27.76]
**Overall**	106,638	18.02 (15.47)	29.31 (15.76)	10.70 (8.91)	15.72 (7.55)
		13.21 [7.46, 23.06]	27.14 [17.28, 39.21]	8.08 [4.51, 14.07]	16.46 [8.92, 21.58]
**PH = 180 min**					
**Meals ($\Pi_{M}$)**	68,825	24.03 (18.52)	39.59 (9.67)	14.40 (11.15)	24.41 (4.40)
		18.60 [11.20, 31.11]	37.76 [33.31, 43.42]	11.17 [6.48, 19.01]	24.10 [22.65, 24.55]
**Night ($\Pi_{N}$)**	6800	11.49 (10.59)	16.14 (7.44)	7.61 (5.16)	9.10 (2.80)
		8.30 [5.71, 13.40]	13.66 [10.40, 19.19]	5.88 [4.02, 9.72]	8.74 [7.46, 10.62]
**Hypo treatment ($\Pi_{H}$)**	663	33.35 (25.15)	42.76 (26.38)	20.56 (15.94)	25.95 (14.90)
		25.14 [15.71, 40.97]	29.77 [21.70, 66.61]	16.13 [10.11, 25.79]	21.81 [13.20, 33.91]
**Overall**	76,288	22.99 (18.40)	32.83 (20.20)	13.85 (11.00)	19.82 (11.71)
		17.46 [10.29, 29.79]	29.77 [17.89, 39.22]	10.60 [6.08, 18.24]	18.07 [10.32, 24.55]
**PH = 240 min**					
**Meals ($\Pi_{M}$)**	47,763	25.60 (19.12)	36.80 (10.52)	15.19 (10.76)	23.54 (4.84)
		20.18 [12.57, 32.58]	36.84 [30.45, 41.36]	12.18 [7.35, 19.99]	23.21 [19.46, 26.55]
**Night ($\Pi_{N}$)**	1176	11.79 (10.20)	15.41 (7.35)	7.94 (5.55)	9.09 (3.60)
		8.39 [6.26, 12.90]	12.32 [11.41, 19.31]	5.96 [4.46, 9.39]	7.86 [6.84, 11.47]
**Hypo treatment ($\Pi_{H}$)**	203	46.01 (32.58)	42.30 (40.44)	26.50 (18.78)	28.76 (24.81)
		32.35 [21.02, 68.73]	27.17 [15.67, 62.29]	18.23 [11.87, 36.15]	22.90 [11.61, 38.15]
**Overall**	49,142	25.36 (19.19)	29.84 (22.94)	15.06 (10.80)	19.19 (14.37)
		19.89 [12.28, 32.26]	24.65 [13.00, 37.04]	12.01 [7.21, 19.82]	16.25 [8.29, 25.32]

**Table 7:** Prediction error of different literature methods with in silico studies. Acronyms: ANN (artificial neural network), NN-PLA (neural network plus linear prediction algorithm), LVX (latent variable-based model), PM (physiological model), CRNN (convolutional recurrent neural networks), DRNN (dilated recurrent neural networks), CHO (carbohydrates), $R_{a}$ (glucose rate of appearance from a meal). * data for the adult cohort.

Study	Method	Inputs	Virtual Cohort	#Days	PH (min)	${\mathrm{RMSE}}_{\mathrm{last}}$ (mg/dL)
Daskalaki et al. [24]	ANN	CGM and	10 adults +	8	30	2.8 *
		insulin infusion	10 adolescents + 10 children		45	4.0 *
Zecchin et al. [25]	NN-LPA	CGM, CHO,	20 subjects	11	30	9.4
		predicted $R_{a}$				
Zhao et al. [26]	LVX	CGM, insulin	10 adults	7	30	8.6
		infusion, and CHO			60	14.0
Liu et al. [27]	PM		10 adults	14	30	10.90
		CGM, insulin			60	24.44
		infusion, and CHO			90	33.50
					120	37.63
Li et al. [28]	CRNN	CGM, insulin	10 adults	360	30	9.38
		infusion, and CHO			60	18.87
Zhu et al. [29]	DRNN	CGM, insulin	10 adults	360	30	7.8
		infusion, CHO,				
		and sampling time				

## Data Availability

The simulated datasets used in this study are available on request from the corresponding author.

## Abbreviations

The following abbreviations are used in this manuscript:

T1D	Type 1 Diabetes
CGM	Continuous Glucose Monitoring
IDF	International Diabetes Federation
MARD	Mean Absolute Relative Difference
SMBG	Self-Monitoring Blood Glucose
PG	Plasma Glucose
CG	Capillary Glucose
